# Comparison of Auto- and Fixed-Continuous Positive Airway Pressure on Air Leak in Patients with Obstructive Sleep Apnea: Data from a Randomized Controlled Trial

**DOI:** 10.1155/2019/6310956

**Published:** 2019-08-07

**Authors:** Marius Lebret, Marie-Caroline Rotty, Cyril Argento, Jean-Louis Pepin, Renaud Tamisier, François Arbib, Dany Jaffuel, Nicolas Molinari, Jean-Christian Borel

**Affiliations:** ^1^HP2, INSERM U1042, Grenoble Alpes University, Grenoble, France; ^2^AGIR à dom. Association, Meylan, France; ^3^IMAG, CNRS, Montpellier University, Montpellier University Hospital, Montpellier, France; ^4^APARD Groupe Adène, Montpellier, France; ^5^Sleep Laboratory, Thorax and Vessels Division, Grenoble Alpes University Hospital, Grenoble, France; ^6^Cabinet de Pneumologie, Échirolles, France; ^7^Department of Respiratory Diseases, Montpellier University Hospital, Arnaud de Villeneuve Hospital, Montpellier, France; ^8^Pulmonary Disorders and Respiratory Sleep Disorders Unit, Polyclinic Saint-Privat, Boujan sur Libron, France; ^9^Department of Medical Information, Montpellier University Hospital, Montpellier, France

## Abstract

Auto-CPAP may cause sleep fragmentation due to variations in pressure and unintentional leaks. The aim of this study was to compare air leak between fixed-CPAP and auto-CPAP after 4 months of CPAP treatment. This study is an ancillary analysis of a randomized, double-blind, parallel, controlled trial over 4 months, comparing fixed- and auto-CPAP in newly diagnosed patients with OSA. The following data were extracted from the CPAP devices: mean and 90th percentile pressure, residual apnea-hypopnea index, mean CPAP use, and amount of leak. Within each arm, patients were also randomly allocated to use of one of the three different brands of devices. Since the leak was reported differently for each device, median leak value was determined for each brand and leaks were classified as “above the median” or “below the median”. Data from 269 patients were analyzed. The univariate analysis showed that tobacco consumption, CPAP level, and oronasal masks were associated with leaks above the median value but not the type of CPAP. The multivariate analysis showed that only CPAP level and oronasal masks were associated with leaks below the median. There were no differences in the types of mask used between fixed- and auto-CPAP. There was no impact of the type of CPAP on leaks or the type of interface used. We used a method based on the median leak value to standardize comparisons across devices which report leaks with different definitions.

## 1. Introduction

CPAP is the first-line treatment for moderate to severe obstructive sleep apnea (OSA) syndrome [1, 2]. The most frequently reported side effect of CPAP is leaks [3, 4] that cause annoyances such as mouth dryness and nose congestion [5, 6]. The etiology of leaks is multifactorial [7], and over the past decades, manufacturers have striven to improve interfaces and CPAP algorithms in order to attenuate leaks and reduce mean pressures during sleep. Autoadjusting CPAP is an important technological advancement that adapts the positive pressure applied according to residual obstructive respiratory events (flow limitation or hypopnea/apnea) that are constantly detected by the device. This has the advantage of maintaining upper airway patency whatever the body position or sleep stage [8]. Auto-CPAP was initially used for pressure titration [9] but is now largely deployed for long-term CPAP treatment [10]. Auto-CPAP has a similar level of effectiveness as fixed-CPAP in terms of normalization of the apnea-hypopnea index (AHI), with a significantly lower mean pressure applied during the night and a slightly higher rate of compliance [11].

In the AgirSASadom study, a large randomized controlled trial (RTC) (*n* = 322) that compared the impact of autoadjusting CPAP versus fixed-CPAP on blood pressure (BP) in OSA, auto-CPAP did not reduce 24 h diastolic blood pressure as efficiently as fixed-CPAP [12] This might be due to microarousals caused by variations in therapeutic pressures during sleep [13, 14] as well as unintentional leakage [15, 16] caused by sudden increases in therapeutic pressure in response to respiratory events. In a previous systematic review [7], we investigated the impact of CPAP mode (auto- versus fixed-CPAP) on leak. We found six randomized controlled trials (RCTs) that reported data about the amount of leak from both auto-CPAP and fixed-CPAP, five of which found no difference between these modes [10, 17–20]. However, although they reported that data, the studies were not specifically designed to address that issue and the sample sizes were small (from 10 to 80 patients).

The primary objective of the present study was therefore to compare the level of leak between fixed-CPAP and auto-CPAP after 4 months of CPAP treatment. Given the importance of the type of mask on air leak [21, 22], and since the influence of the type of CPAP on the type of mask used has never been assessed, the secondary objective was to compare the proportions of nasal and oronasal masks used in each group. For this purpose, we carried out an ancillary analysis of the AgirSASadom study mentioned above [12].

## 2. Materials and Methods

### 2.1. Data Source

The database from the AgirSASadom study was used and approval was granted by the ethics committee, *Comité de Protection des Personnes Sud-Est V*, Grenoble, France (REC No: 09-AGIR-2), and registered on clinicaltrials.com (NCT01090297). All participants provided signed informed consent for their participation. The AgirSASadom study was a single-centre, randomized, double-blind, parallel, controlled trial carried out over 4 months and designed to compare the effectiveness of fixed- and auto-CPAP in reducing BP in newly diagnosed patients with OSA. An extensive description of this study can be found elsewhere [12].

### 2.2. Participants

Patients with OSA aged 18–80 years for whom CPAP therapy was indicated were eligible. Patients were recruited from June 2010, and follow-up was completed by October 2012.

### 2.3. Randomization and Intervention

Patients who met the eligibility criteria were randomized to receive either fixed- or auto-CPAP. Within each arm, patients were also randomly allocated to be provided with one of the three brands of CPAP devices (Philips, WEINMANN, and ResMed). All patients were initiated to CPAP by experienced nurses who followed the same protocol: all patients were systematically fitted with a nasal mask on initiation to CPAP; however, an oronasal mask could be provided in case of major difficulty breathing with the nasal mask during a diurnal trial or during the follow-up. Optimal therapeutic pressure was titrated by one expert researcher based on the 90th percentile pressure. In the auto-CPAP group, the minimum width of the window of pressure variation was 5 cmH_2_0. At the 4-month visit, the type of interface (nasal or oronasal), use of a heated humidifier, and use of a chin strap were documented. The following data were also extracted from the CPAP devices: mean pressure (cmH_2_O), 90th percentile pressure (cmH_2_O), residual apnea-hypopnea index (events/h), mean CPAP use (hours/night), and leak. The different measurements of leak provided by the built-in software of each device are presented in Table 1.

### 2.4. Data Collection and Processing

This study involved three different brands of CPAP devices. We were thus faced with the issue of different definitions and methods of reporting leak between brands (Table 1). In order to be able to compare levels of leak [23], the median leak value was determined for each brand and leaks were classified accordingly as “above the median” or “below the median,” regardless of CPAP mode.

**Figure 1:**
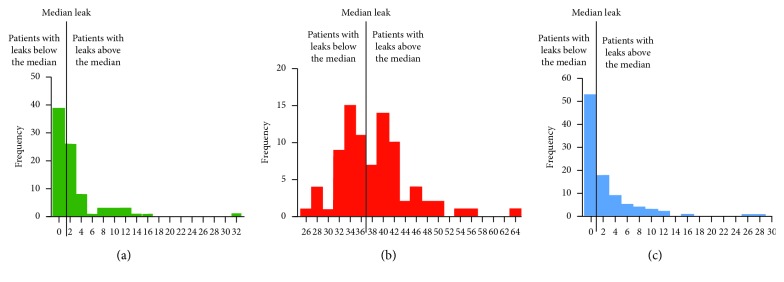
Classification of patients according to leakage reported by machines; median leak value was determined for each brand. (a) Distribution of leak magnitudes in the ResMed devices expressed as unintentional leakage (L/min) (*x*-axis). (b) Distribution of leak magnitudes in the Philips devices expressed as unintentional leakage (L/min) (*x*-axis). (c) Distribution of leak magnitudes in the WEINMANN devices expressed as unintentional leakage (L/min) (*x*-axis).

Figure 1 shows the distribution of levels of leak for each brand, with their median values. Further details regarding these methods are provided in the supplemental file (available here).

### 2.5. Statistical Analysis

The data did not follow a Gaussian distribution; therefore, continuous data are expressed as medians and interquartile ranges (IQR). Qualitative parameters (characteristics and type of interface) were expressed as counts and percentages.

#### 2.5.1. Achieving the First Objective

Univariate logistic regression models were used to estimate the association between leaks that were above the median and the following variables: age, body mass index, initial AHI, active smoking, hypertension, diabetes, residual AHI (estimated by CPAP built-in software), Epworth Sleepiness Scale (ESS), type of mask, chin strap, heated humidifier, CPAP use, CPAP level, and type (fixed versus auto). Then, using a stepwise selection, covariates with a *P* value <0.15 in the univariate analysis were fed into a multivariate model. The OR and 95% CI were calculated according to Woolf's method, with an alpha risk of 0.05. The goodness of fit of this model was assessed using the Hosmer–Lemeshow test.

#### 2.5.2. Achieving the Second Objective

A chi-square test was used compare to the proportion of nasal and oronasal masks used according to CPAP mode.

Other differences in patient characteristics between fixed- and auto-CPAP were tested using a chi-squared test for categorical data and a Mann–Whitney–Wilcoxon test for continuous data. All statistical analyses were performed with SAS Enterprise Guide (V.7.1).

## 3. Results

**Figure 2:**
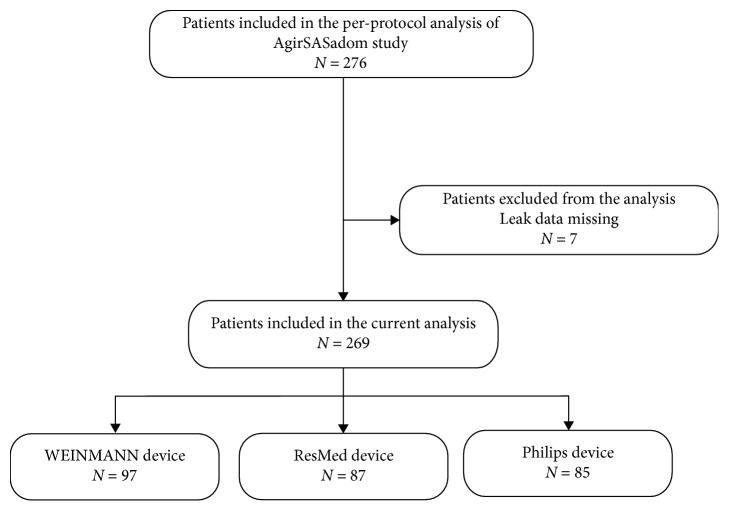
Study flowchart: patients included in the analysis.

Of the 276 patients included in the per-protocol analysis of the AgirSASadom study, leak data were missing for 7, and thus, they were excluded from the analysis (*n* = 4 in the auto-CPAP group); data from 269 patients were therefore included in the analysis (Figure 2). Data regarding anthropometric characteristics, comorbidities, treatment parameters, and sleep apnea severity at diagnosis are presented in Table 2.

### 3.1. Determining Factors Associated with Large Leaks

In the univariate analysis, active smoking, mean pressure level, and use of an oronasal mask were positively associated with a risk of leaks above the median in the whole population (Table 3). Type of CPAP was not associated with a risk of leaks above the median (primary objective). In the multivariate analysis, use of an oronasal mask and mean pressure level remained independently associated with a higher risk of leaks above the median.

The results of a subanalysis based on the manufacturers' recommended thresholds for unintentional leakage are provided in the supplemental file (available here).

### 3.2. Use of Nasal versus Oronasal Masks according to Type of CPAP

Table 4 shows the comparison of CPAP and interface-related parameters between patients with auto-CPAP and fixed-CPAP after 4 months of treatment. There was no significant difference in the type of mask, use of a chin strap, or heating humidifier between groups. Mean pressure level was significantly higher in the fixed-CPAP group.

## 4. Discussion

This is one of the largest studies to compare the type of CPAP (fixed versus auto) on leaks. The results showed that there was no association between the type of CPAP and the risk of leaks above the median leak level. Leak was mainly dependent on the type of interface and mean pressure, irrespective of the type of CPAP.

To our knowledge, this is the first study to evaluate the impact of the type of CPAP on leak across different brands of devices. We thus had to overcome the issues relating to the different methods of defining and reporting of leak between manufacturers, which prevented direct comparison of data. Moreover, this is confusing in routine clinical practice [23]. The “acceptable” leak thresholds proposed by manufacturers are arbitrary and are not related to clinical outcomes. Moreover, the amount of leak required to affect the effectiveness of CPAP therapy and patient compliance remains unknown [23]. We thus proposed a method to compare leak data from different brands of CPAP devices, using the median leak value as a threshold for each brand. Another solution might have been to analyze the extreme values (e.g., 90th and 10th percentile), which may have been more clinically meaningful. However, this would have meant using only the data from patients with extreme leak values and would have reduced the size of the sample analyzed. Finally, using the definitions of the “acceptable” leak thresholds proposed by the manufacturers to compare leak data between brands was not feasible because of the selection bias introduced by the per-protocol analysis. Indeed, at the 4-month follow-up visit, the sample included demonstrated good compliance with CPAP (5.5 (3.9; 6.7) hours/night) and only 4 patients experienced leakage above the manufacturer leak thresholds.

The fact that auto-CPAP was not associated with larger leaks confirms the results of other studies that involved smaller samples [7]. Although those studies were not specifically designed to address the issue of leak, no differences in leak were found between fixed- and auto-CPAP, despite lower median pressures delivered by auto-CPAP [7]. The present results also showed that the type of CPAP did not influence mask selection. Therefore, although this analysis was not part of the outcome measures of the original study and constitutes a post hoc analysis, neither leaks nor interfaces contributed to the difference in 24 h diastolic blood pressure between autoadjusting and fixed-CPAP found in the study by Pépin et al. [12].

This study highlighted some factors related to leak. First, the level of pressure was positively associated with a risk of leaks above the median leak level. This is well known by clinicians and concordant with our previous results that identified high levels of CPAP as an independent factor relating to unintentional leak [24]; this reinforces the validity of the method used in the present study to analyze leak. Second, the oronasal mask was also an independent contributor to leak. The ability of oronasal masks to control unintentional leaks remains controversial [7, 24]: several previous studies comparing oronasal versus nasal interfaces showed larger leaks with oronasal masks [17, 21, 25, 26]. Although in clinical practice they are commonly fitted to overcome suspected mouth leakage, our results highlighted that oronasal masks may not be an effective strategy to reduce leak in the overall population.

Finally, active smoking was significantly linked to leak rate in the univariate analysis. This interesting result is likely due to the higher level of nasal resistance caused by the increase in inflammatory response with tobacco consumption [27, 28]. This is a key factor in the persistence of mouth opening and mouth breathing under CPAP [29, 30], favoring mouth leak. It could be hypothesized that smokers were therefore more likely to be fitted with an oronasal mask, which may explain why this variable was no longer significant in the multivariate analysis (confounding factor).

## 5. Conclusions

In conclusion, the present analysis showed that the type of CPAP did not affect the amount of leak, using an original method to standardize the definition of leaks across different brands of devices. In addition, the type of interface used by patients was not influenced by the mode of CPAP. However, the measurement and reporting of leaks remain confusing, both for clinicians and in clinical trials. Manufacturers must make an effort to standardize the reporting of leaks, as recommended in the American Thoracic Society statement [23]. Further studies are needed to determine if there is a threshold leak level above which there is a negative impact on the effectiveness of CPAP treatment.

## Figures and Tables

**Table 1:** Leak data reported for each device brand and CPAP mode.

Type of device	Definition of leak data	Fixed-CPAP group	Auto-CPAP group
WEINMANN	Time spent with large leakage expressed in %	0.4 (0.0; 2.4)	1.5 (0.1; 4.5)

ResMed devices	Median nonintentional leakage expressed in L/min	1.2 (0.0; 2.4)	1.2 (0.0; 3.6)
	95th percentile of nonintentional leakage expressed in L/min	12.0 (6.0; 21.6)	10.8 (6.0; 16.8)

Philips devices	Mean total leakage expressed in L/min	39.6 (35.2; 41.5)	36.0 (33.5; 40.2)
	90th percentile of total leakage expressed in L/min	47.9 (42.0; 51.1)	45.1 (41.0; 49.5)

Data are expressed as medians (25th; 75th) and means (SD). Device names: SOMNOsmart 2 for WEINMANN, S8 Spirit 2 for ResMed, and Remstar Auto for Philips. This table shows how the 3 manufacturers report leakage for each mode of PAP used in the study: fixed or auto. Data reporting differ between each of the CPAP adherence tracking system devices.

**Table 2:** Patient characteristics.

Anthropometric data and comorbidities	General population (*n* = 269)	Fixed-CPAP (*n* = 130)	Auto-CPAP (*n* = 139)	*p* value
Age (years)	58 (50; 64)	58 (51; 65)	57 (49; 64)	0.51
Sex, female, No. (%)	76 (28.3)	36 (27.7)	40 (28.8)	0.84
BMI (kg/m^2^)	30.1 (27.0; 34.8)	31.2 (27.5; 35.1)	29.6 (26.6; 34.5)	0.11
Current smoker, No. (%)	51 (19.0)	25 (19.2)	26 (18.7)	0.91
Hypertension, No. (%)	180 (66.9)	95 (73.1)	85 (61.2)	0.04
Diabetes, No. (%)	49 (18.2)	25 (19.2)	24 (17.3)	0.68

*Treatment parameters*				
Residual AHI (events/h)	3.7 (2.2; 6.9)	3.8 (2.2; 8.0)	3.6 (2.1; 6.3)	0.31
CPAP use (hours)	5.5 (3.9; 6.7)	5.7 (3.9; 6.8)	5.4 (3.8; 6.7)	0.41
CPAP level, (cmH_2_O)	8.5 (7; 10)	9.0 (8; 11)	7.5 (6.5; 9.4)	<0.0001
Oronasal mask^a^, No. (%)	99 (37.9)	52 (41.9)	47 (34.3)	0.20
Chin strap, No. (%)^b^	5 (1.9)	0 (0)	5 (3.6)	0.06
Heated humidifier, No. (%)^c^	130 (48.7)	64 (50)	66 (47.5)	0.68

*Sleep apnea severity at diagnosis*				
AHI, (events/h)	39.4 (29.6; 56.9)	39.4 (29.0; 59.1)	39.8 (30.0; 56.6)	0.62
Epworth sleepiness scale, 1–24	9 (6; 13)	10 (6; 15)	9 (6; 13)	0.07

BMI: body mass index, AHI: apnea-hypopnea index, and CPAP: continuous positive airway pressure; data are expressed as % or median (IQR). ^a^Mask type from 8 patients was missing; ^b^chin strap use from 6 patients was missing; ^c^heated humidifier use from 5 patients was missing. Data are reported as medians and quartiles or numbers and percentages, as appropriate.

**Table 3:** Logistic regression analysis with “leaks above the median value” as the dependent variable.

	Univariate analysis			Multivariate analysis		
	OR	95% (CI)	*P* value	AOR	95% (CI)	*P* value
Age (years)	1.01	(0.99; 1.03)	0.37			
Sex, female	0.78	(0.46; 1.32)	0.35			
BMI (kg/m^2^)	1.02	(0.98; 1.05)	0.41			
Initial AHI (event/h)	1.01	(0.99; 1.02)	0.36			
Current smoker	2.10	(1.12; 3.93)	**0.02**			
Hypertension	0.98	(0.59; 1.63)	0.93			
Diabetes	1.42	(0.76; 2.64)	0.27			
Residual AHI (event/h)	1.05	(0.99; 1.11)	0.10			
CPAP use (hours)	0.95	(0.84; 1.07)	0.38			
Fixed-CPAP	1.17	(0.73; 1.89)	0.52			
CPAP level (cmH_2_O)	1.25	(1.11; 1.41)	**0.0003**	1.19	(1.05; 1.35)	**0.0084**
Oronasal mask	2.75	(1.64; 4.61)	**0.0001**	2.23	(1.30; 3.83)	**0.0037**
Chin strap	0.71	(0.12; 4.35)	0.71			
Heated humidifier	1.18	(0.73; 1.90)	0.51			

BMI: body mass index, AHI: apnea-hypopnea index, CPAP: continuous positive airway pressure, OR: odds ratio, and AOR: adjusted odds ratio. Data are reported as medians and quartiles or numbers and percentages, as appropriate.

**Table 4:** Comparison between fixed- and auto-CPAP.

	Fixed-CPAP	Auto-CPAP	*P* value
*Device*			
Current AHI (event/h)	3.7 (2.2; 8.0)	3.6 (2.1; 6.5)	0.38
CPAP use (h/night)	5.7 (3.9; 6.7)	5.3 (3.8; 6.7)	0.35
CPAP level (cmH_2_O)	9.0 (8.0; 11.0)	7.5 (6.5; 9.4)	**<0.0001**

*Interface*			
Oronasal^a^	52 (41.6)	49 (35.3)	0.29
Chin strap^b^	0 (0)	6 (4.23)	NA
Heated humidifier^c^	64 (49.6)	67 (47.2)	0.69

AHI: apnea-hypopnea index and CPAP: continuous positive airway pressure. Data are expressed as % or median (IQR); ^a^mask type from 8 patients was missing; ^b^chin strap use from 6 patients was missing; ^c^heated humidifier use from 5 patients was missing.

## Data Availability

The data used to support the findings of this study are available from the corresponding author upon request.

## Supplementary Materials

Supplementary Materials The section “Leakage algorithm” presents the mathematical methods used to classify the level of leak for each brand with their median values. The section “Subanalysis: risk of leakage above the manufacturer thresholds” presents the results of a subanalysis based on the manufacturer's recommended thresholds for unintentional leakage.
