# Supplementary material for: Comparison of Auto- and Fixed-Continuous Positive Airway Pressure on Air Leak in Patients with Obstructive Sleep Apnea: Data from a Randomized Controlled Trial
Source: Can Respir J. 2019 Aug 7;2019:6310956. doi: 10.1155/2019/6310956 (PMC6702837; doi:10.1155/2019/6310956)
Supplement: Supplementary Materials — The section “Leakage algorithm” presents the mathematical methods used to classify the level of leak for each brand with their median values. The section “Subanalysis: risk of leakage above the manufacturer thresholds” presents the results of a subanalysis based on the manufacturer's recommended thresholds for unintentional leakage. [file 6310956.f1.docx]

**Supplementary File**

**Leakage algorithm**

In order to compare leak data expressed in l/min across device brands, new categorical variables (V’) were created (see algorithm below) based on the median leak measured by each brand. In the case of values tied exactly at the median, a related variable (Vbis), which was an estimate of the associated 90/95-percentile, was considered. In case of missing l/min data (e.g. the device in question did not produce l/min data), we applied the same algorithm to percentage data.

If V_i_ is expressed in l/min then;

If V_i_ < median (V) then V’_i_ = “below”;

Else if V_i_ > median (V) then V’ _i_ = “above”;

Else if Vbis_i_ < median (Vbis) then V’ _i_ = “below”;

Else if Vbis_i_ > median (Vbis) then V’ _i_ = “above”;

Else V’ _i_ = random (“below”, “above”).

Else if V_i_ is expressed in percentage then;

If V_i_ < median (V) then V’ _i_ = “below”;

Else if V_i_ > median (V) then V’ _i_ = “above”;

Else V’ _i_ = random (“below”, “above”).

Where Vi (resp Vbisi) is an individual value of a device-recorded variable and V'i (resp V'bisi) is the individual value of the new categorial variable. V=(V1, ..., Vn) and Vbis=(Vbis1, ..., Vbisn).

**Subanalysis: risk of leakage above the manufacturer thresholds**

1. **Methods and sample**

A logistic regression model was used to model the risk of leakage above the manufacturer thresholds.

**Patients treated with the Resmed device (N =87):**

Thirteen patients had leaks within the 95th percentile > 24 L/min (Resmed threshold)

(7 under fixed-CPAP vs 6 under auto-CPAP).

**Patients treated with the Philips device (N=**85)

Leak was reported by Philips in L/min: if we consider a threshold of 60 L/min (the threshold reported in Schwab et al., 2013 for total leak), 7 patients had leaks within the 90th percentile, above 60 L/min (4 under fixed-CPAP vs 3 under auto-CPAP).

**Patients treated with the Weinmann device (N=97)**

No threshold is reported in L/min by Schwab et al. for the Weinmann device but if we extrapolate the threshold of one hour with large leakage reported in Schwab et al. (20% of a standard night of 5 hours – the median nightly use of CPAP in our study), 2 patients using a Weinmann device had high leakage (0 under fixed-CPAP vs 2 under auto-CPAP).

In total, 22 of the 269 patients had leakage above the respective manufacturer thresholds.

1. **Results**

|  | Univariate analysis :  Risk of leakage above the manufacturer thresholds | | |
| --- | --- | --- | --- |
|  | OR | 95% [CI] | P-value |
| Fixed CPAP group | 0.73 | [0.30; 1.76] | 0.48 |
